# Energy Starved *Candidatus* Pelagibacter Ubique Substitutes Light-Mediated ATP Production for Endogenous Carbon Respiration

**DOI:** 10.1371/journal.pone.0019725

**Published:** 2011-05-09

**Authors:** Laura Steindler, Michael S. Schwalbach, Daniel P. Smith, Francis Chan, Stephen J. Giovannoni

**Affiliations:** 1 Department of Microbiology, Oregon State University, Corvallis, Oregon, United States of America; 2 Department of Zoology, Oregon State University, Corvallis, Oregon, United States of America; Argonne National Laboratory, United States of America

## Abstract

Previous studies have demonstrated that *Candidatus* Pelagibacter ubique, a member of the SAR11 clade, constitutively expresses proteorhodopsin (PR) proteins that can function as light-dependent proton pumps. However, exposure to light did not significantly improve the growth rate or final cell densities of SAR11 isolates in a wide range of conditions. Thus, the ecophysiological role of PR in SAR11 remained unresolved. We investigated a range of cellular properties and here show that light causes dramatic changes in physiology and gene expression in *Cand.* P. ubique cells that are starved for carbon, but provides little or no advantage during active growth on organic carbon substrates. During logarithmic growth there was no difference in oxygen consumption by cells in light versus dark. Energy starved cells respired endogenous carbon in the dark, becoming spheres that approached the minimum predicted size for cells, and produced abundant pili. In the light, energy starved cells maintained size, ATP content, and higher substrate transport rates, and differentially expressed nearly 10% of their genome. These findings show that PR is a vital adaptation that supports *Cand.* P. ubique metabolism during carbon starvation, a condition that is likely to occur in the extreme conditions of ocean environments.

## Introduction

A decade has passed since Beja et al. first identified [1] and characterized [2] proteorhodopsin (PR) genes from uncultivated bacterioplankton, showing that PR functioned as a proton pump when cloned and expressed in *E. coli*. Since then, a large number of molecular surveys have confirmed that PR genes are ubiquitous in bacteria throughout the marine photic zone [3]–[10]. Bacteria expressing heterologous PR from a marine bacterium of the uncultured SAR86 clade were shown to benefit from the pumping activity of the PR protein under starvation conditions: *E. coli* expressing PR increased flagellar motility in the light when cellular respiration was inhibited [11], and *Shewanella oneidensis* expressing PR increased viability in the light under nutrient limited conditions [12].

Although most evidence has supported an energetic role for PR as a proton pump, in the few studies that have been done with cultivated marine bacteria a consistent physiological function for PR has not emerged. Light-induced differences in growth [13], bicarbonate uptake [14], and survival to starvation [15] were observed in three marine bacterial species. *Dokdonia* sp. strain MED134 was shown to grow better in the light than in darkness, especially when grown in low concentration of dissolved organic matter [13], *Vibrio* sp. strain AND4 was shown to have increased long-term survival when starved in seawater exposed to light rather than when held in darkness [15]. But in other species, both growth and expression of the PR gene were independent of light exposure [16]. For example, the enhancement of growth in the light that was observed in one *Dokdonia* species [13] was not observed in another member of the genus, *D. donghaensis* strain PRO95 [16], but the absence of a stimulatory effect of light on the growth of this latter strain may also result from the higher concentrations of carbon used for its growth. *Candidatus* Pelagibacter ubique was the first example of a cultured marine bacterium harboring the PR gene, and also the first example in which no enhancement of growth or difference in PR gene expression was observed in cells exposed to light [17]. Years of work with *Cand.* P. ubique in culture corroborated the early report that light does not impact the growth of these cells in batch cultures grown in a variety of conditions (examples in Figure S1).

*Cand.* P. ubique is a member of the SAR11 clade of alpha-Proteobacteria, which is now widely accepted as the most abundant group of heterotrophic bacteria in the oceans. They represent approximately one quarter of all rRNA genes identified in clone libraries from marine environments [18], and, according to fluorescence *in situ* hybridization cell counts, one third of the prokaryotic cells in the surface waters of the northwestern Sargasso Sea [19]. Hence, determining the role of PR in this key marine bacterium is essential for understanding the ecological roles of PR broadly.

The objective of this study was to test hypotheses about the function of PR in *Cand.* P. ubique, strain HTCC1062 by studying the impact of light on a range of cellular properties, including respiration rates, ATP-dependent substrate transport rates, ATP content per cell, cell morphology and whole genome gene expression patterns. The data rule out the hypothesis that light enhances growth efficiency and instead implicate PR in a complex cellular response to energy limitation that includes light-dependent suppression of endogenous respiration in carbon-limited cells.

## Results

### Respiration experiment

Our initial hypothesis was that PR enables a more efficient use of organic carbon in the light than the dark, but without a change in growth rate, by enabling cells to meet their energy requirements while respiring less carbon per cell division. To test this hypothesis we compared the relationship between respiration and biomass production across exponential and stationary phases of bacterial growth in the light and the dark. To attain an exponential phase and a stationary phase that mimicked carbon replete and depleted conditions respectively, we devised a growth medium (described in Materials and Methods) that would achieve the high cell densities (10^7^–10^8^ cells/ml) required for performing non-invasive O_2_ measurements (using optode sensors), but where cells would enter stationary phase as a consequence of carbon limitation.

Cell counts and dissolved oxygen were measured in parallel HTCC1062 cultures grown either in continual darkness or light∶dark cycles (12∶12 hr duration). Irradiance was of ca. 250–300 µmol photons m^−2^ s^−1^.

**Figure 1:**
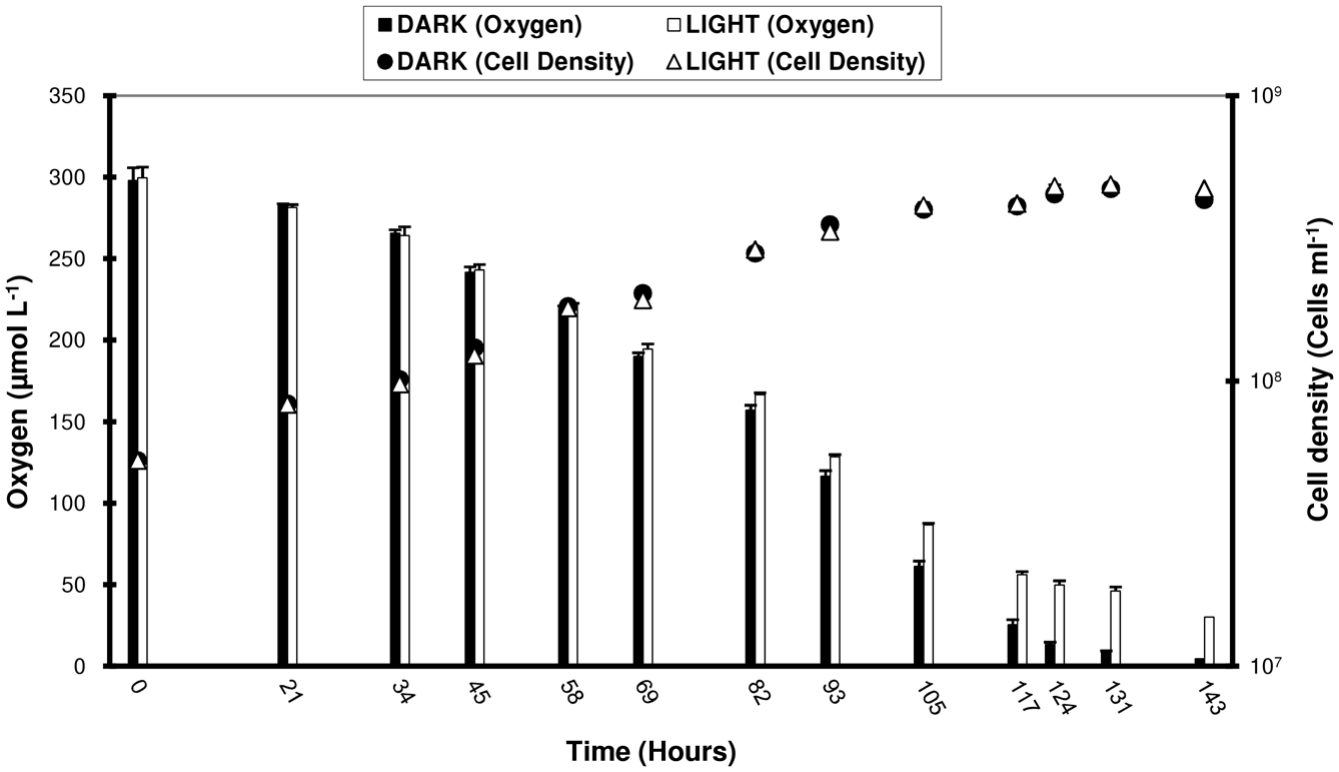
Respiration decreases in the light compared to the dark as organic carbon becomes limiting. Cell density and oxygen concentration of HTCC1062 grown under continuous darkness (filled circles, black bars) or light∶dark (12∶12 hr cycles) (open triangles, white bars) during logarithmic and stationary growth phase. The light source was a mixture of cool white and GRO-LUX fluorescent light bulbs, the intensity was of ca. 250–300 µmol photons m^−2^ s^−1^. Cells were grown in glass air-tight sealed bottles (transparent for light treatments or opaque for dark) that were submerged in a 17°C water bath in order to maintain constant temperature.

Growth rates and maximum cell densities were the same in the dark and in the light (Figure 1). Dissolved oxygen was depleted at similar rates in the light and dark during early and mid-logarithmic growth phase, i.e. as long as cells were not limited in carbon. These results indicated that during active growth, the relationship between *Cand.* P. ubique biomass accumulation and organic carbon oxidation was unaltered by light. However, in late exponential phase and stationary phase, the rate of respiration decreased in the light as compared to the dark as the availability of organic carbon became limiting (Figure 1).

### Microscopic analysis of the morphology of cells

**Figure 2:**
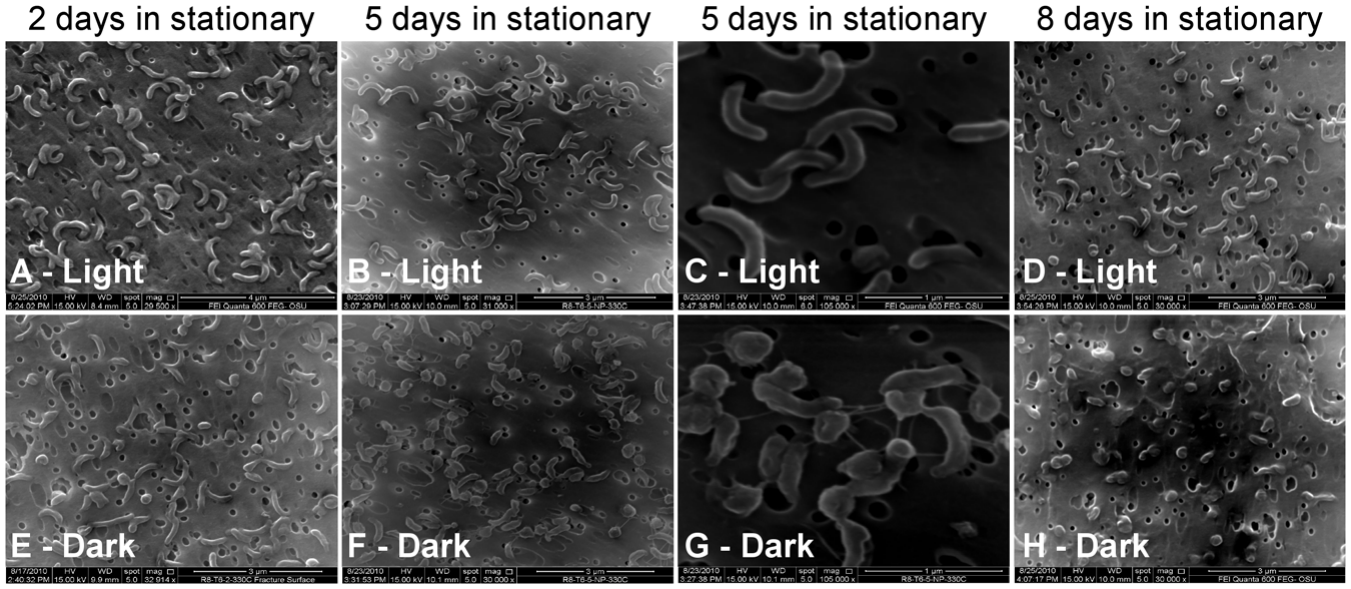
Under carbon-limiting conditions, cells in the light are able to maintain their morphology, while cells in darkness became smaller. Scanning electron microscopy of HTCC1062 cells grown either in continuous light, 70 µmol photons m^−2^ s^−1^ (A, B, C, D) or in darkness (E, F, G, H), two, five and eight days after entering stationary phase. Pictures on day five were taken at two different magnifications (∼30000× (B and F) and 105000× (C and G)); higher magnification was used to show the pili formed by dark grown cells. Original high resolution pictures are available on Supporting Information.

We investigated the impact of light on cell morphology by sampling HTCC1062 cells grown in either light (70 µmol photons m^−2^ s^−1^) or dark during exponential and stationary phases for analysis by scanning electron microscopy. We found that during exponential growth, *Cand.* P. ubique morphology in light and dark conditions was the same - i.e. the comma shape morphology that is typical of these organisms (as in Figure 2A). However, once in stationary phase, cells in the light maintained the comma shape (Figure 2 B–D), while cells grown in dark became smaller (Figure 2F–H). Interestingly, we observed pili connecting cells in cultures that had been in stationary phase and complete darkness for 5 days (Figure 2G). Each individual SEM image from Figure 2 is provided as a high resolution image in Figure S4, Figure S5, Figure S6, Figure S7, Figure S8, Figure S9, Figure S10, Figure S11.

### Measurements of ATP contents per cell

**Figure 3:**
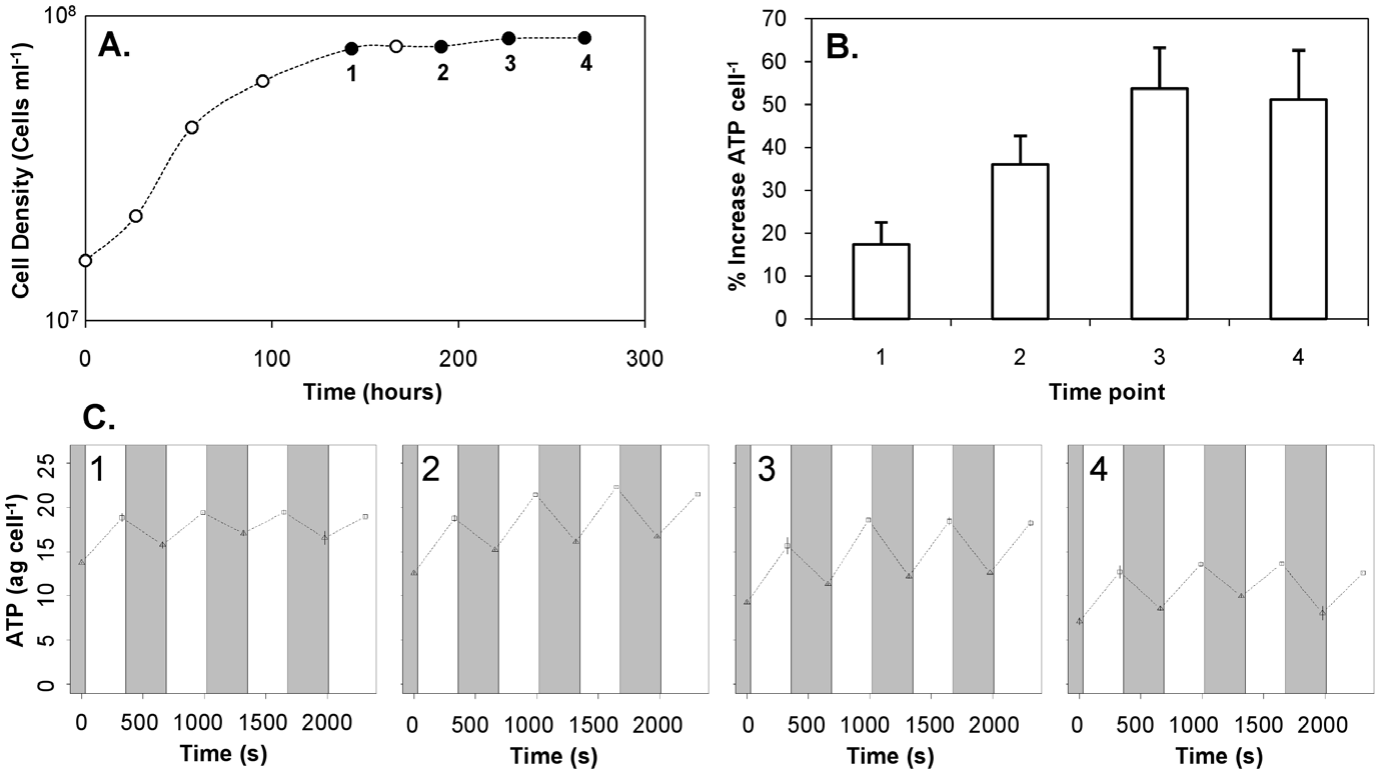
PR contributes a higher percentage to the total energetic budget of the cells as the period of starvation lengthens. Sequential measurements of cellular ATP content performed over 35 min, including 4 dark to light shifts. A) growth curve of HTCC1062 cells showing the four time points of the ATP assays (filled black dots). B) ATP content/cell (mean±range of duplicate samples) five minutes after exposure to either dark (triangles, grey background) or light (squares, white background) at the four time points shown in A. C) Percent increase in ATP/cell as a result of light exposure (mean±s.d. of the last three dark to light shifts in each graph). The light source, utilized during the dark-light shifts, was a white lamp covered by a green filter that transmits light mainly in the 500–580 nm wavelength range at ca. 70 µmol photons m^−2^ s^−1^.

To experimentally test for light-dependent production of ATP, we measured the cellular ATP after five minute intervals of light (70 µmol photons m^−2 ^s^−1^) or dark exposure in cultures in early stationary phase and 2, 3, and 5 days after entering stationary phase (Figure 3A). Our results showed that ATP increased 17.4±5.1% after 5 minutes light exposure compared to 5 minutes dark exposure in the early stationary phase sample and increased by 36.1±6.7%, 53.7±9.6% and 51.1±11.6% two, three and five days later, respectively (Figure 3B, C). We then determined the added % ATP content/cell in dark to light shifts for cells grown in a richer medium (10× more pyruvate, oxaloacetate and taurine) with cells from logarithmic and late logarithmic phase. Results from this experiment showed that, after light exposure, the increase in ATP/cell was marginal during exponential growth, 6.3±3.4%, but increased to 11.6±2.1% in late logarithmic phase (Figure S2).

### Taurine uptake efficiency in the light versus darkness

**Figure 4:**
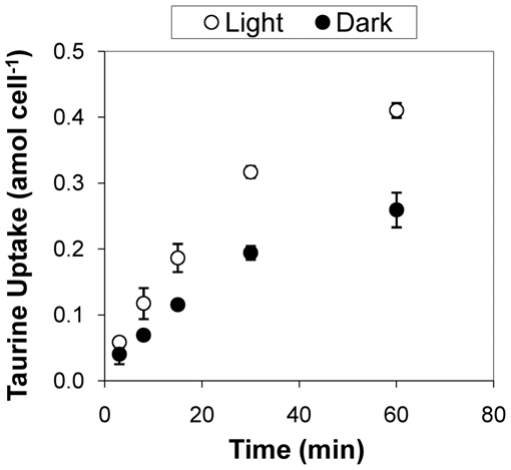
Taurine-uptake transport capacity of starved *Candidatus* Pelagibacter ubique cells is 65% more efficient in the light than in darkness. Uptake of radio-labeled ^14^C-taurine (1 µM final concentration) by HTCC1062 cells in light, (80 µmol photons m^−2^ s^−1^, open circles) versus dark (closed circles; mean±s.d. of triplicate measurements). Assays were conducted at room temperature (22°C) in artificial seawater media.

The efficiency of ATP-dependent transport into starved HTCC1062 cells in light (80 µmol photons m^−2^ s^−1^) versus dark was tested utilizing ^14^C-labled-taurine. Taurine was chosen for this assay because the HTCC1062 genome contains the genes *tauABC* (GeneID: 3516719-21) which are predicted to encode an ATP-dependent taurine transporter, and also because taurine was previously shown to be utilized for growth by HTCC1062 [20]. Results from radio-labeled assays determined that the uptake rate of taurine was 65% higher in the light than in the dark (Figure 4).

### Global gene expression patterns in Cand. P. ubique in the light versus darkness

Affymetrix GeneChip oligonucleotide microarrays for HTCC1062 were used to measure changes in transcription caused by illumination. In an early experiment we had compared the transcriptional response in light versus dark grown HTCC1062 cells harvested during logarithmic phase, but found few significant differences (NCBI GEO database, reference number GSE24134). Given the results described above, which indicated a physiological effect of light only in carbon starved conditions, we suspected that a transcriptional response to light might be evident only in carbon limited cells. Thus we compared the transcription profiles in stationary phase cells grown on 12∶12 hrs light∶dark cycles (light treatment, irradiance of 250 µmol photons m^−2^ s^−1^ produced by a green LED device) and cells grown exclusively in dark (dark treatment). Almost one tenth of coding sequences (9.7%) were found to be differentially expressed between treatments; 4.7% up-regulated in the light treatment (n = 64) and 5% up-regulated in the dark treatment (n = 68) (Table S1). Comparisons of the transcription profiles of cells from the light treatment, with the same cells that were transferred to darkness for 6 hours (light-to-dark treatment), showed an overlapping, but much smaller change in genetic expression (Table S2).

Three transcriptional regulators, SAR11_0964 (3516571), SAR11_1242 (3517798), and SAR11_0138 (3516752), were upregulated in the light treatment as compared to the dark treatment, and may be involved in regulating the expression of genes that were differentially expressed: (Table S1). Two of these regulators, SAR11_0964 (3516571) and SAR11_1242 (3517798), were also downregulated, 2.7 and 1.8 fold respectively, in cells from the light-to-dark treatment (Table S2).

One of these transcriptional regulators, SAR11_0964 (3516571) codes for a putative ferric uptake regulator protein (Fur). Fur is known to be a global regulator that binds to sequences, known as Fur-boxes, found upstream of the regulated genes [21]. Studies of *E. coli* originally led to the proposal that the Fur-box was a 19-bp inverted repeat sequence [22], but alternatively the Fur-box has also been viewed as a head-to-head-to-tail repeat of a simple hexamer [23], or also as two overlapping heptamer inverted repeats [(7-1-7)_2_] that together define a 21-bp sequence [24]. Bioinformatic searches using the three Fur box models on the HTCC1062 genome gave no positive results. More relaxed searches that included variants of these models resulted in several interesting motif sequences found upstream of the transcriptional regulators (SAR11_0964 (3516571) and SAR11_1242 (3517798)) and other five genes that were differentially expressed in the light versus dark treatments (Figure S3). These sequences, found using sequence-motif searches, are different from classical fur-boxes in other bacteria, thus experiments are needed to confirm whether they indeed are regulatory sequences recognized by Fur proteins or by other transcriptional regulators.

Several components of the oxidative phosphorylation pathway were upregulated in the dark: components of the cytochrome *bc1* complex (*fbcH* and *petB* (3516594-5)), components of the cytochrome *c* oxidase (*ctaB*, *cox1* (3516747-9)), and components of the ATP-synthase complex (*atpB*, *atpE*, *atpB* (3516768-70), *atpD*, *atpG*, *atpA*, *atpH* (3517558-61)) (Table S1). These results imply a higher rate of oxidative phosphorylation in the dark than in the light and support the faster depletion of oxygen observed in carbon starved dark grown cells. In the dark we also observed the up-regulation of a proton-translocating pyrophosphate synthase (SAR11_1040 (3517439)), which may be an additional mechanism to produce energy for the starved cells in the dark.

The microarray analysis revealed that the pilin gene (*pilA* (3516629)) was up-regulated in the dark treatment. Microscopy showed that starved cells grown in the dark not only became smaller, but eventually produced pili that connected cells.

In mixed cultures (transcriptomes of microcosms and of environmental samples) PR has been reported to be more highly expressed during periods of daylight [25], [26]. Surprisingly, proteorhodopsin in our axenic HTCC1062 culture was found to be expressed more highly (2 fold) in the dark than in the light during stationary phase. Genes involved in protein folding and stabilization under stress (*groES*, *groEL* (3516888-9)) were also upregulated in the dark. Metabolic pathways and transport systems were also differentially expressed in the light and the dark. Under dark growth conditions there was a prevalence of amino acid transport systems, e.g. the *yhd* operon (*yhdW*, *yhdX*, *yhdY*, *yhdZ* (3516962-3, 3516561-2), *tauA*, from the taurine transport system (3516721), and *livJ*, *livJ2*, *braC* (3517043, 3517459 and 3516881): three genes annotated as related to amino acid transport, while in the light, there was an up-regulation of genes involved in glycolysis and the glyoxylate cycle (*fbaB*, *pgk*, *gap* (3517096-8); *gyaR*, *glcFED* (3517602-6); *mrsA* (3517115)). In the light, two genes related to recombination and DNA repair (*xerD* (3517456) and *recA* (3516867)) were also up-regulated.

Complete lists of the differentially expressed genes are given in Table S1 and Table S2. A change of at least 2 fold (for single genes) or an average change of ≥1.7 fold, for operons (p≤0.05) was considered significant. The microarray data files have been deposited in the NCBI GEO database, reference number GSE24134.

## Discussion

The discovery of proteorhodopsins in ocean metagenomes had a major impact because it demonstrated a new conduit by which light energy might enter the biosphere. An altered perspective of food webs emerged in which cells that were ostensibly heterotrophs were postulated to improve their carbon assimilation efficiency by adopting some of the qualities of phototrophs. While effects of light were demonstrated for some bacteria, for many, including *Cand.* P. ubique, the function of PR remains controversial because the predicted differences in growth rates or cell yields between dark and light cultures were not observed [16], [17], [27]. Uncertainty about the role of PR was not resolved by studies of gene transcription, which showed that PR is expressed constitutively in relatively large amounts in logarithmically growing cells (GSE24134), but that light causes no change in protein content or gene expression. In this study we explored the function of PR by measuring respiration rates, cellular ATP contents, transport capacities and cell morphology. These data reveal that in *Cand.* P. ubique PR plays a powerful role in a complex cellular response that maintains cell functions during periods of carbon starvation. In the context of the new data, previous reports of constitutive expression of the *Cand.* P. ubique PR in actively growing cells emerges as a red herring which was misleading because it suggested a function for PR in actively growing cultures. In retrospect, the constitutive expression of the *Cand.* P. ubique PR may instead reflect strong selection for an immediate response to cellular energy deficits caused by carbon starvation.

Metagenomic data indicate that PRs are highly diverse and are distributed widely among microbial taxa in the oceans [3]–[10]. It has been suggested that diverse rhodopsins could potentially have a wide range of physiological functions, e.g. as light activated proton pumps, or as sensory proteins involved in signaling [27]. Cultured bacteria harboring PR genes are also phylogenetically diverse, and encompass copiotrophic species such as *Dokdonia* sp. [13], [16], which can readily replicate on high concentrations of organic carbon substrates, as well as oligotrophic strains, such as *Cand.* P. ubique [17] and SAR92 [28], which are typically more abundant in seawater but cannot be cultivated on organically rich media. This considerable variation may explain why a uniform response to light has not been observed among cultured strains containing PR genes. In *Dokdonia* sp. strain MED134, PR can provide energy not only for respiration and maintenance functions, but also for active growth [13]. On the other hand, no increase in growth rate or cell yields was observed in the light for SAR11 and SAR92 bacterial clades [17], [28], which appear to be more highly adapted to oligotrophic conditions.

We chose *Cand.* P. ubique for this study because the SAR11 clade, to which it belongs, indisputably plays an important role in ocean surface ecology, and thus is a good model to understand how PR and light influence microbial community function of the oceans. The *Cand*. P. ubique PR has been shown to function as a proton pump when cloned into *E. coli*, is present in large amounts that are less likely explained by a sensory function, has rapid photocycling rates characteristic of ion pumps rather than sensory rhodopsins, and has conserved amino acids that are appropriately positioned to act as proton acceptor and donor residues in the retinylidene Schiff-base transfer reaction [17]. The new evidence we reported here, showing an increase in ATP levels and transport rates in the light, and a decrease is respiration, further supports the conclusion that PR in *Cand*. P. ubique functions as a light-driven proton pump rather than as a sensory molecule involved in signal transduction.

One of the most attractive theories for PR function is that it replaces respiration, thereby allowing cells to use scarce organic carbon resources more efficiently [13]. This theory predicts a higher ratio of dissolved organic carbon consumed to biomass produced by heterotrophic consumers and thus has important consequences for the carbon cycle. We used oxygen uptake as a proxy for carbon oxidation and found that oxygen depletion occurred at the same rate in the light and in the dark as long as cells were not limited in carbon, arguing against the theory that *Cand.* P. ubique cells could replace the respiration of exogenous organic carbon with a light-driven proton pump when growing exponentially (Figure 1). However, unexpectedly, we observed that respiration decreased considerably in the light compared to the dark as the availability of organic carbon became limiting, indicating that PR exerted its impact only when cells became carbon limited.

The higher rates of respiration in dark-grown cells entering stationary phase suggested that they were consuming endogenous reserves to produce ATP for survival. We tested this hypothesis by microscopic analysis and found that indeed *Cand.* P. ubique cells were consuming their biomass when starved in the dark, while in the light they could maintain their size. *Cand.* P. ubique is among the smallest autonomously replicating cells known, with a volume ranging from 0.019 to 0.039 µm^3^ (as extrapolated from our electron microscopy measurements). It has one of the smallest genomes known for free living bacteria (only 1,308,759 base pairs), and a very slow growth rate (∼one division in two days), which enables lower requirements for ribosomes, and thus such small cell volumes. Here we found that *Cand.* P. ubique, when starved in the dark, consumes its endogenous reserves and shrinks to a volume of ∼0.014 µm^3^. This extremely small volume is very close to that predicted for the theoretical minimal-size microbe, which was proposed to be in the range of 0.005–0.01 µm^3^ [29]. Constraints on the lower limits of the size of a free-living prokaryote may be imposed by factors such as the number of protein and RNA species required for minimal essential functions, the size of the genome required to encode these essential macromolecules, the number of ribosomes necessary for adequate expression of this genome, and physical contraints, such as the minimum radius of curvature required for the stability of a lipid bilayer membrane [29].

Starved *Cand.* P. ubique cells kept in the light were able to maintain their morphology presumably due to ATP production from light energy. Although it has been assumed that PR-induced proton motive force could drive ATP synthesis as protons reenter the cell through the ATP synthase complex [1], [2], this hypothesis has heretofore only been tested in a heterologous system [30]. We observed that ATP content per cell increased in stationary phase cells after they were illuminated in proportion to the length of time since entering stationary phase. Thus, PR contributed a higher percentage to the total energetic budget of the cells as the period of starvation lengthened. These results support the conclusion that cells maintain their size and morphology in the light under starved conditions by replacing endogenous respiration with PR-mediated ATP production.

The ability to produce ATP in absence of exogenous carbon sources is likely to be particularly important to the survival of *Cand.* P. ubique because a large proportion of its nutrient transporters belong to the ATP-binding cassette family [31], and thus require ATP hydrolysis for the import of their specific substrates. Proteome measurements with both cultured SAR11 cells and environmental samples from the Sargasso Sea and the Oregon Coast showed very high expression of ABC-transport systems in SAR11 cells [32]–[34]. Increases in ATP production and transport functions such as those we observed in illuminated, carbon starved *Cand.* P. ubique cells may confer a valuable survival advantage by enabling them to rapidly restart respiratory metabolism when oxidizable organic compounds again become available in the environment. This would be in agreement with the function of PR in *Vibrio* sp. strain AND4, where enhanced survival was observed in cells exposed to light compared to darkness during starvation [15].

The necessity of producing ATP to restart metabolism may explain why carbon-starved *Cand.* P. ubique cells in the dark continue to respire endogenous carbon until their size approaches an extreme minimum. However, this finding raises questions about how light causes the suppression of endogenous respiration. We observed that transcription profiles in carbon-starved *Cand.* P. ubique changed significantly when the cells were illuminated. Whether transcriptional changes in cells in the light were mediated directly or indirectly is unknown, but clearly light potentiated a genetic response, with nearly one tenth of coding sequences being differentially expressed. Three transcription regulators that were found to be upregulated in the light may be involved in these transcriptional changes, but this can currently not be confirmed due to the absence of a genetic system in *Cand*. P. ubique.

One of the transcriptional regulators up-regulated (7.4 fold) in the light, SAR11_0964 (3516571), belongs to the ferric uptake transcription regulator gene-family. The Fur protein is a global regulator that usually acts as a negative regulator of iron acquisition genes, but it has also been implicated in the regulation of other cellular processes, such as energy metabolism, and specifically respiratory electron transport [35]. This was suggested by comparisons of the transcriptional profiles of wild type and *fur* mutants of *Shewanella oneidensis*, where it was found that genes involved in energy metabolism were differentially expressed [35], [36]. Fur proteins bind to specific sequences (Fur boxes), but no classic Fur box sequences were found in the *Cand.* P. ubique genome (Accession number NC_007205.1). Less stringent bioinformatic searches of inverted repeat motifs found upstream of genes that were differentially expressed suggested the possibility of an energy regulon in *Cand.* P. ubique that is potentiated by Fur, but future experimental work is needed to validate these results.

Some specific changes in gene expression appeared to be in accord with the observed physiological responses to light. For example, genes for oxidative phosphorylation were more highly expressed in the dark. The pili were detected during starvation in darkness and higher expression of the pilin gene was observed under the same conditions. The role of pili in starved *Cand.* P. ubique is not known, but similar pilin genes have been implicated in nucleic acid uptake in other organisms [37]. Previous work on multiple SAR11 strains from a single population revealed one of the highest recombination rates recorded in bacteria [38], [39]. We speculate that the expression of pili during starvation conditions could underlie these high rates of recombination.

Cells also appear to express metabolic pathways differently while carbon starved in the light, up-regulating genes involved in glycolysis and the glyoxylate cycle, while cells in the dark up-regulated genes for amino acid transport. In a metatranscriptomic study comparing microbial communities during the day and at night, Poretsky and collaborators also found evidence indicating that amino acid acquisition may be a more important metabolic activity at night [26]; the authors suggested that accumulation of amino acids at night might be a mechanism for nitrogen storage. Different regulation of mechanisms of uptake and metabolic pathways in the light and dark may be related to the different compounds that are released from phytoplankton, and thus available to bacteria, during day and night. Daytime is also when light induced DNA damage occurs, which may explain the observed upregulation of DNA-repair mechanisms in the light (*xerD* and *recA*). The upregulation of *hflC* in the dark, on the other hand, may be related to the proteolytic activity involved in the oxidation of endogenous carbon in starved cells grown in the dark.

A decade has elapsed since proteorhodopsin was first reported, and many chapters remain unwritten in the story of how this protein influences ocean biology. This report focuses attention on a role for PR as one component of a complex, systemic response of cells to a survival stress that is likely to be common in the oceans - carbon starvation. Future research may scrutinize how cells enter and leave the minimized state, and determine whether light exerts its influence on transcription by a direct mechanism, or indirectly, by sensing the cell's ATP level. Given the role of SAR11 as an example of a highly abundant ocean heterotroph, it will also be of interest to examine how PR and cellular systems associated with it contribute to the success of SAR11 populations in the environment.

## Materials and Methods

### Growth conditions

Seawater for experiments was collected at Newport Hydroline station NH-05 (latitude: 44.65°, longitude: −124.18°) from 10 meters depth between July, 2009 and April, 2010. Water was transported to the laboratory and filtered through a 0.2 µM Supor (Millipore) membrane prior to being autoclaved. Autoclaved seawater was subsequently sparged first with 0.1 µm filtered CO_2_ and then with 0.1 µm filtered air for a period of 8 hours and 24 hours respectively. In all experiments, autoclaved, sparged seawater media was amended with excess amounts of (final concentrations): nitrogen (NH_4_Cl, 1 mM), inorganic phosphate (KH_2_PO_4_, 100 µM), iron chloride (FeCl_3_, 1 µM) and excess vitamins (final concentrations): thiamine (593 nM), niacin (227 pM), B12 (74 pM), para-amino benzoic acid (PABA, 409 pM), pyridoxine (59 nM), pantothenic acid (81 nM), inositol (555 nM). To this base seawater media, pyruvate, oxaloacetate, taurine, betaine, glycine and methionine were added in a treatment-dependent manner, described below. Assays were conducted in either 250 ml polycarbonate flasks, 10 L polycarbonate carboys, or glass bottles, and were incubated at either 17 or 22°C (depending on experiment) in continuous darkness, continuous light, or in light∶dark (12∶12 hr) cycles for the duration of the experiments. Light intensities were measured with a quantum reader (Biospherical Instruments QSL-100) fitted with a spherical quantum sensor. Cells were quantified by staining with the DNA stain SYBR Green I (Invitrogen) for one hour, followed by enumeration with a flow cytometer (Guava Technologies) [40].

### Respiration measurements

Preliminary growth curves were performed to identify a growth medium that satisfied three conditions: 1) reaching high cell densities in order to be able to measure oxygen consumptions with the non-invasive method used; 2) stationary phase had to be a result of carbon depletion; 3) cells had to reach stationary phase prior to becoming oxygen limited. Therefore titrations of three carbon sources (pyruvate, oxaloacetate and taurine) yielded the conditions that satisfied these three requirements (data not shown), and the cells were grown in the following medium: autoclaved filtered seawater amended with pyruvate (80 µM), oxaloacetate (40 µM), taurine (40 µM), betaine (1 µM), glycine (50 µM), methionine (50 µM), iron source (1 µM), vitamins (as described in the Growth Condition section). The culture was first grown in a 10 L carboy until it reached a cell density of 5.3×10^7^ cells/ml. Then it was passed into twenty-eight 170 ml glass transparent bottles (that had been first acid washed, rinsed and autoclaved). Half of the bottles were darkened completely by being covered with black electric tape. Bottles were completely filled with culture and sealed air-tight. To eight of the bottles (4 transparent ones and 4 darkened ones) an optode sensor spot (PreSens Precision Sensing GmbH, Germany) was inserted inside the bottle in order to enable non-intrusive oxygen measurements with the oxygen measuring instrument, Fibox 3-trace (PreSens Precision Sensing GmbH, Germany). Oxygen measurements from the light and dark treatment replicates were taken at time intervals of 10–14 hours. The other bottles (the ones not containing the optode sensor spot) were used for cell counts. Every time oxygen measurements were taken, two bottle replicates of each light and dark treatment were used for the cell counts. Measurements were taken until cultures reached stationary phase (ca. 4.7×10^8^ cells/ml) and for a couple more days. All the bottles were kept in a single water bath that circulated water at 17°C. Light (a mixture of cool white and GRO-LUX fluorescent light bulbs) was turned on and off in a 12∶12 hr cycle, and the intensity (reaching cultures in transparent bottles) was of ca. 250–300 µmol photons m^−2^ s^−1^.

### Scanning Electron Microscopy

HTCC1062 cells used for morphological analysis were grown in the same medium as for the respiration experiment, in either continuous light (70 µmol photons m^−2^ s^−1^) or continuous darkness. Subsamples of cells were harvested two, five and eight days after the original cultures had entered stationary phase, and were fixed in the original growth medium with 0.5% glutaraldehyde, for at least 1 h at 4°C. Samples were then filtered on 13 mm polycarbonate 0.2 µm membrane filters (Nuclepore™ track-etched polycarbonate membranes, Whatman) using a Swinnex-13 Filter Holder (Millipore) and rinsed in a graded series of ASW with distilled water (75%, 50%, 25%, 0% ASW) for 5 minutes each; then dehydrated in a graded series of ethanol with distilled water (10%, 25%, 50%, 75% ethanol) for 10 minutes each. Just before critical point drying, samples were passed to ethanol 100% for 10 minutes. Critical point drying was performed with liquid CO_2_, after which samples were sputter coated with gold for 35 seconds and mounted on stubs for examination at 15 kV in a field emission scanning electron microscope (Quanta 600 FEG, Oregon, USA). Original Pictures taken on the SEM and utilized for creating Figure 2 are available in Figure S4, Figure S5, Figure S6, Figure S7, Figure S8, Figure S9, Figure S10, Figure S11.

### ATP measurements

Cultures for ATP measurements were grown in the same medium as for the respiration experiment in a light∶dark (12∶12 hrs) cycle. The light source during growth of HTCC1062 cells in this experiment was green light-emitting diode (LED, Aqua Illumination) at an intensity of ca. 250 µmol photons m^−2^ s^−1^. Samples for measurements were taken at different stages of growth (early stationary and three later stationary phase time points) for ATP assays. An additional culture where cells were grown in a richer growth medium (containing 10 times more pyruvate, oxaloacetate and taurine) was used for the same assay performed on cells during logarithmic growth. The assay was performed as follows: 3 ml of cell suspension were placed in 5 ml glass vials. A water bath was used to minimize heat transfer from the light source to the samples. ATP was measured by using a luciferase-based assay (BactTiter Glo, Promega, Madison, WI) as follows. At each time point, 2 aliquots (20 µl each) of the sample were dispensed into white 96-well assay plates (White w/Lid, Tissue Culture-Treated, BD Biosciences, San Jose, CA). Ninty microliters of BactTiterGlo reagent were added per well, and luminescence was measured after 4 min using a multi function plate reader (Infinite M200, Tecan) with a 1 s integration and 10 ms settle time. An ATP standard curve was used to calculate the concentration of ATP in the samples. The light source, utilized during the dark-light shifts, was a white lamp covered by a green filter (medium green filter 660, GamColor, Los Angeles, CA) that transmits light mainly in the 500–580 nm wavelength range. Irradiance under these conditions was ca. 70 µmol photons m^−2^ s^−1^. Every 5.5 minutes the light was turned on or off, and 5 minutes after each light or dark period samples were taken for ATP measurements.

### Radio tracer experiment

*Candidatus* P. ubique strain HTCC1062 cells utilized for radio tracer assays were cultivated under 12∶12 hrs light∶dark cycles in autoclaved filtered seawater amended with pyruvate (100 µM), oxaloacetate (50 µM), taurine (50 µM), betaine (1 µM), glycine (25 µM), methionine (25 µM), iron source (1 µM), vitamins. Cells were harvested when cultures had entered stationary phase (carbon starved) and collected via centrifugation (1 hour at 20000 rpm) at 16°C in polycarbonate centrifuge tubes. Following centrifugation, the supernatant was poured off and cells were washed twice in minimal-salt artificial seawater mix pH 8.2 (ASW, comprised of NaHCO_3_ (6 mM), NaCl (481 mM), MgSO_4_ (28 mM), MgCl_2_ (27 mM), CaCl (10 mM), KCl (9 mM) HEPES (pH 7.5, 1.0 mM) [41], all chemicals were obtained from Sigma-Aldrich. Then cells were resuspended in ASW and transferred to four 15 ml polystyrene centrifuge tubes that had been pre-rinsed in triplicate with ASW prior to use for radio assays. Two tubes were exposed to light (80 µmol photons m^−2^ s^−1^), and two to dark (covered in aluminum paper), one of each served as negative control (dead cells, preserved with formalin, see below).

Taurine-L-[^14^C] (1 µM, final concentration) radio assays were conducted at room temperature (ca. 22°C). Radioisotopes were obtained from American Radiolabeled Chemicals Incorporated and were supplied in sterile water. Controls were processed in an identical fashion to live samples but were preserved with formalin (2%) for one hour prior to the addition of the isotope to the samples. Determination of uptake of radio-labeled substrate was conducted as follows: 700 µl of cells were collected in triplicates at each time point via filtration through a 25 mm GSVP 0.22 µm filter (Millipore) and filters were then rinsed 6 times with 2 ml aliquots of ASW. After rinsing, all filters were transferred to scintillation vials containing 5 ml of UltimaGold (Perkin-Elmer) scintillation fluid and allowed to sit overnight prior to being read on a Beckman LS-65000 liquid scintillation counter.

### Microarray experiments

HTCC1062 cells used in microarray experiments were grown in batch cultures (50 ml each in 250 ml polycarbonate flasks). For the comparison of cells collected during logarithmic phase the medium was autoclaved filtered seawater containing 1 µM methionine, 1 µM serine, 10 µM NH_4_, 1 µM PO_4_, 10 nM FeCl_3_ and vitamins, and cells were grown in either 24 hrs dark or 24 hrs light. 40 ml of culture from each biological replicate were harvested via centrifugation (1 hour at 20 000 rpm, 10°C), then re-suspended in 1 ml of Bacteria Protect (Qiagen) for 15 min.

For the comparison of transcription profiles from cells in stationary phase, the growth medium used was the same as for the respiration experiment. Cells were grown in a 17°C growth chamber either in darkness, “dark treatment”, (flasks covered in aluminum paper), or in light∶dark (12∶12 hrs) cycles, “light treatment”, where light (250 µmol photons m^−2^ s^−1^) was produced by a green LED device (Aqua Illumination). Growth assays were conducted in triplicate for each treatment. At cell-harvesting time, after cells had reached stationary phase (time = 270 hours), 10 ml of culture samples from each replicate culture of the light treatment and the dark treatment were harvested and immediately stabilized using 20 ml of RNA stabilization reagent (RNAprotect Bacteria reagent, Qiagen). In addition, an aliquot (10 ml) from each replicate of the cells from the light treatment was transferred to darkness for 6 hours before harvest, “light-to-dark treatment”, and then RNA was stabilized the same way as the other two treatments. Following RNA stabilization, cells were collected by centrifugation (30 min at 40 000 g, 10°C), the supernatant was removed, and cells were then stored frozen at −80°C prior to extraction. RNA for microarrays was collected using RNeasy Mini kits (Qiagen) and amplified and biotin-labeled using the MessageAmp-II Bacteria RNA amplification kit (Ambion) per the manufacturer's instructions; 10–15 ng of template RNA was used per reaction. Resulting amplified and labeled RNA (aRNA) was screened for length and quality using a Bioanalyzer 2100 (Agilent) and quantified utilizing a Nanodrop 1000 spectrophotometer (Thermo Fisher Scientific). Five µg of biotinylated aRNA from triplicate samples was fractionated and then hybridized (45°C) overnight to custom ‘*Candidatus* Pelagibacter ubique’ Affymetrix GeneChip arrays that contained probes for HTCC1002, HTCC1062 and HTCC7211 (Pubiquea520471f) using Affymetrix GeneChip Fluidics Station 450, and Affymetrix GeneChip Hybridization Oven 640. Arrays were then washed following manufacturer's instructions and the resulting images were analyzed using an Affymetrix GeneChip Scanner 3000. Background corrections and raw expression values were normalized between chips via application of the robust microarray algorithm (RMA), quantile-normalization and median polish analyses [42] in the Affymetrix Expression Console program using the AGCC software package. Statistical analysis was conducted using the MultiExperiment Viewer (MeV, version 4.2.1, http://www.tm4.org/) [43]. Differences between treatments were deemed significant when mean RMA normalized signal intensities differed statistically (p-value≤0.05) and genes exhibited either a 2 fold change or greater (for single genes) or an average 1.7 fold change or greater for putative operons, between treatments.

Microarray data files are MIAME compliant and raw data was deposited in the NCBI GEO database, reference number GSE24134.

### Genome-wide motif searches

Motif discovery was facilitated using an iterative scanning algorithm. The search space was limited to nucleotides encompassing the −100 to +50 regions of all genes which were observed to be differentially transcribed during the light or dark treatment. These 366 sequences were scanned for two types of motifs: overlapping inverted repeats and tandem + inverted repeats. Overlapping inverted repeats were discovered by first locating all individual inverted repeats composed of a repeating oligo of between six and 15 nucleotides connected by a spacer that was shorter than or equal to the length of the repeating oligo. Low complexity repeating oligos – those lacking a C or G – were discarded, as were oligos that were better represented by a longer oligo. Tandem + inverted repeats were defined as a repeating oligo of between six and 15 nucleotides which occurred three times in close proximity, separated by spacers of up the length of the repeating oligo. Either the first or last occurrence of the repeating oligo had to be in the opposite orientation (reverse complement) relative to the other two. Matching motifs were similarly required to contain at least one C or G in the repeating oligo.

## Supporting Information

Figure S1 **Light does not affect growth rates or yields in** ***Candidatus*** **Pelagibacter ubique in a wide range of growth conditions.** Nitrogen (NH_4_Cl, 10 µM), inorganic phosphate (KH_2_PO_4_, 1 µM), iron chloride (FeCl_3_, 10 nM), vitamins were added to all the cultures (A–H). A- Autoclaved, filtered seawater amended with glycine (1 µM) and methionine (1 µM). B- Autoclaved, filtered seawater. C and D – Cells were grown in artificial seawater amended with glycine (1 µM), methionine (1 µM) and taurine (2.5 µM); C- Inoculum derived from a logarithmically growing culture; D- Inoculum derived from a stationary phase culture. E- Cells were grown in artificial seawater amended with oxaloacetate (1 µM), methionine (1 µM) and taurine (1.5 µM). F- Same growth conditions as in E but taurine was given at 25 µM. G- Cells were grown in artificial seawater amended with glycine (0.1 µM), methionine (0.1 µM) and taurine (1.5 µM). H- Cells were grown in artificial seawater amended with glycine (0.01 µM), methionine (0.01 µM) and taurine (1.5 µM). Light in the light treatments was given as 12∶12 hrs light dark cycles. Light sources and intensities: A, B, E, F, G, H: fluorescent light, 30 µmol photons m^−2^ s^−1^. C and D: LED green light, 80 µmol photons m^−2^ s^−1^. Means±s.d. of triplicate measurements are given for all curves other than “G” that had a single replicate.
(TIF)

Figure S2 **PR contributes a higher percentage to the total energetic budget of the cells in late-logarithmic growth phase than in mid-logarithmic growth phase.** Sequential measurements of cellular ATP content performed over 35 min, including 4 dark to light shifts. ATP content/cell (mean±range of duplicate samples) five minutes after exposure to either dark (triangles, grey background) or light (squares, white background) using A) cells from logarithmic growth phase, B) cells from late logarithmic phase.
(TIF)

Figure S3 **Potential regulatory sequences upstream of genes differentially expressed in light and dark.**
(TIF)

Figure S4 **Original picture taken with the Scanning Electron Microscope utilized to create** **Figure 2** **.** 2A. Cells after 2 days in stationary phase in the light (70 µmol photons m^−2^ s^−1^). 29500× magnification.
(TIF)

Figure S5 **Original picture taken with the Scanning Electron Microscope utilized to create** **Figure 2** **.** 2B. Cells after 5 days in stationary phase in the light (70 µmol photons m^−2^ s^−1^). 31000× magnification.
(TIF)

Figure S6 **Original picture taken with the Scanning Electron Microscope utilized to create** **Figure 2** **.** 2C. Cells after 5 days in stationary phase in the light (70 µmol photons m^−2^ s^−1^). 105000× magnification.
(TIF)

Figure S7 **Original picture taken with the Scanning Electron Microscope utilized to create** **Figure 2** **.** 2D. Cells after 8 days in stationary phase in the light (70 µmol photons m^−2^ s^−1^). 30000× magnification.
(TIF)

Figure S8 **Original picture taken with the Scanning Electron Microscope utilized to create** **Figure 2** **.** 2E. Cells after 2 days in stationary phase in the dark. 33000× magnification.
(TIF)

Figure S9 **Original picture taken with the Scanning Electron Microscope utilized to create** **Figure 2** **.** 2F. Cells after 5 days in stationary phase in the dark. 30000× magnification.
(TIF)

Figure S10 **Original picture taken with the Scanning Electron Microscope utilized to create** **Figure 2** **.** 2G. Cells after 5 days in stationary phase in the dark. 105000× magnification. Pili connecting cells can be observed.
(TIF)

Figure S11 **Original picture taken with the Scanning Electron Microscope utilized to create** **Figure 2** **.** 2H. Cells after 8 days in stationary phase in the dark. 30000× magnification.
(TIF)

Table S1 **A**) Genes upregulated in cells grown in light∶dark 12∶12 hrs cycles (light treatment). **B**) Genes upregulated in cells grown in constant darkness (dark treatment). Differential expression between treatments was considered biologically relevant for ≥2 fold change for single genes and ≥1.7 fold change for potential operons (t-test, p≤0.05). Genes were considered potential operons if they were consecutive with no (or very small) intergenic space and were either all up or down regulated under light or dark treatment. Question marks (?) represent unknown gene functions. Genes IDs refer to GenBank, SAR11_ #### locus tag is given for unknown genes.
(DOC)

Table S2 **A**) Genes upregulated in cells grown in light∶dark cycles (light treatment). **B**) Genes upregulated in cells from the light treatment transferred to darkness for 6 hours (light-to-dark treatment). Differential expression between treatments was considered biologically relevant for ≥2 fold change for single genes and ≥1.7 fold change for potential operons (t-test, p≤0.05). Genes were considered potential operons if they were consecutive with no (or very small) intergenic space and were either all up or down regulated under light or dark treatment. Question marks (?) represent unknown gene functions. Genes IDs refer to GenBank, SAR11_ #### locus tag is given for unknown genes.
(DOC)
