# Supplementary figures and images for: Energy Starved Candidatus Pelagibacter Ubique Substitutes Light-Mediated ATP Production for Endogenous Carbon Respiration
Source: PLoS One. 2011 May 9;6(5):e19725. doi: 10.1371/journal.pone.0019725 (PMC3090418; doi:10.1371/journal.pone.0019725)

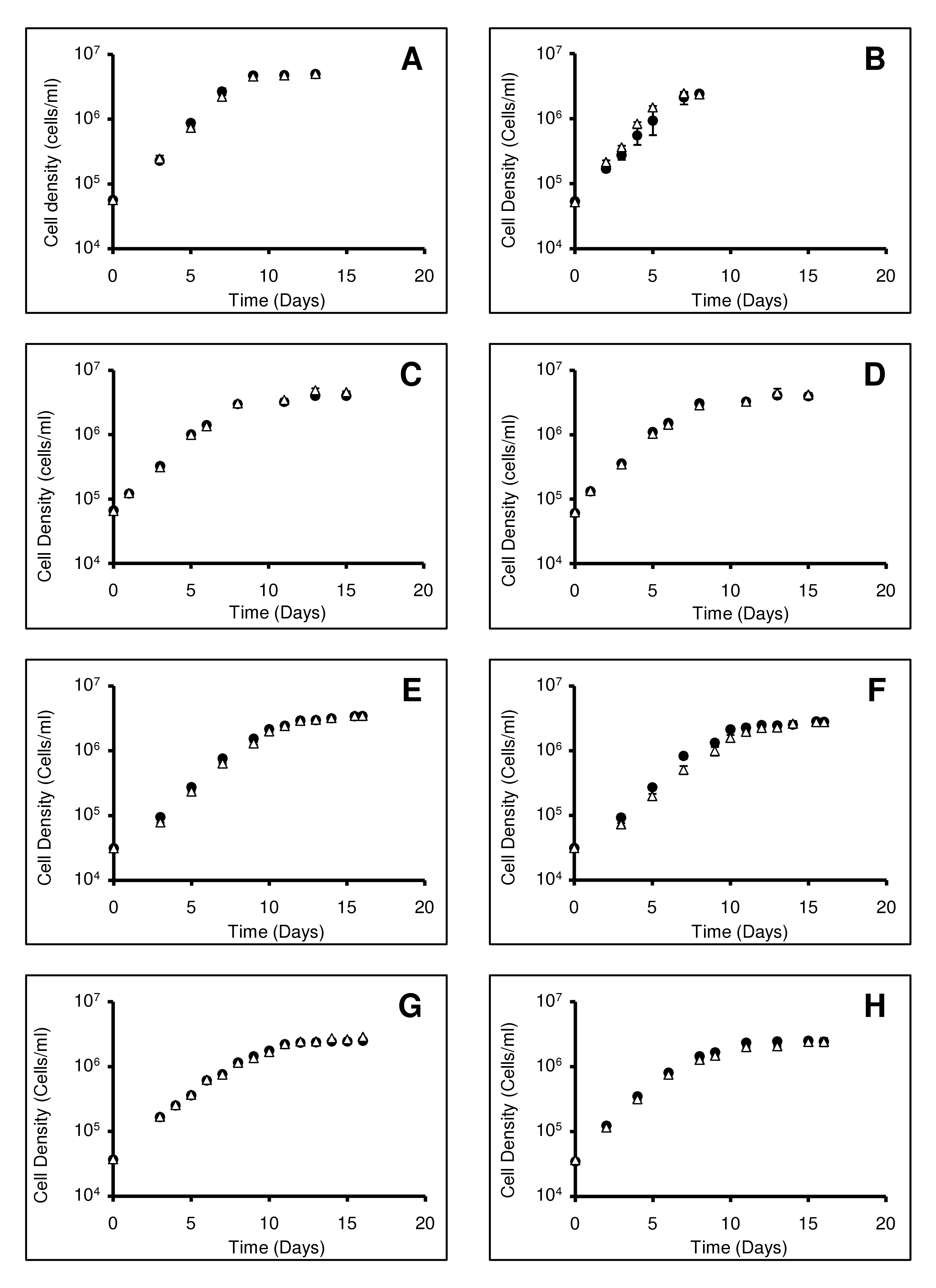

Supplement: Figure S1 — Light does not affect growth rates or yields in Candidatus Pelagibacter ubique in a wide range of growth conditions. Nitrogen (NH4Cl, 10 µM), inorganic phosphate (KH2PO4, 1 µM), iron chloride (FeCl3, 10 nM), vitamins were added to all the cultures (A–H). A- Autoclaved, filtered seawater amended with glycine (1 µM) and methionine (1 µM). B- Autoclaved, filtered seawater. C and D – Cells were grown in artificial seawater amended with glycine (1 µM), methionine (1 µM) and taurine (2.5 µM); C- Inoculum derived from a logarithmically growing culture; D- Inoculum derived from a stationary phase culture. E- Cells were grown in artificial seawater amended with oxaloacetate (1 µM), methionine (1 µM) and taurine (1.5 µM). F- Same growth conditions as in E but taurine was given at 25 µM. G- Cells were grown in artificial seawater amended with glycine (0.1 µM), methionine (0.1 µM) and taurine (1.5 µM). H- Cells were grown in artificial seawater amended with glycine (0.01 µM), methionine (0.01 µM) and taurine (1.5 µM). Light in the light treatments was given as 12∶12 hrs light dark cycles. Light sources and intensities: A, B, E, F, G, H: fluorescent light, 30 µmol photons m−2 s−1. C and D: LED green light, 80 µmol photons m−2 s−1. Means±s.d. of triplicate measurements are given for all curves other than “G” that had a single replicate. (TIF) [file pone.0019725.s001.tif]

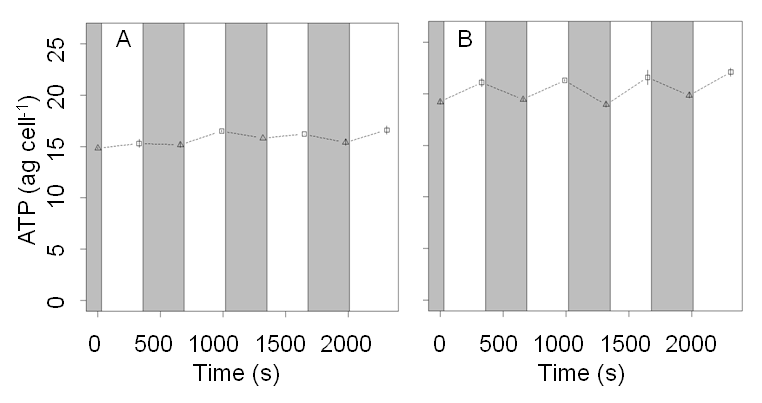

Supplement: Figure S2 — PR contributes a higher percentage to the total energetic budget of the cells in late-logarithmic growth phase than in mid-logarithmic growth phase. Sequential measurements of cellular ATP content performed over 35 min, including 4 dark to light shifts. ATP content/cell (mean±range of duplicate samples) five minutes after exposure to either dark (triangles, grey background) or light (squares, white background) using A) cells from logarithmic growth phase, B) cells from late logarithmic phase. (TIF) [file pone.0019725.s002.tif]

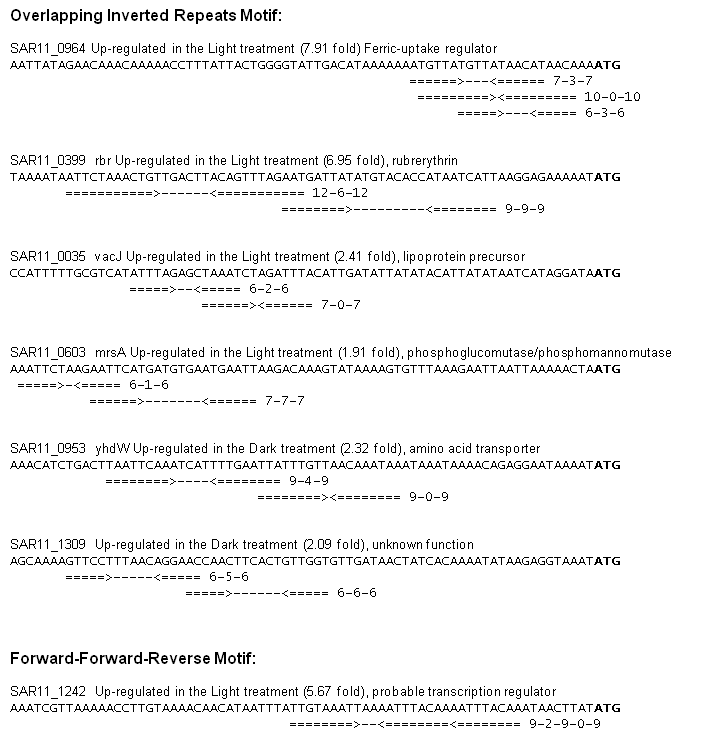

Supplement: Figure S3 — Potential regulatory sequences upstream of genes differentially expressed in light and dark. (TIF) [file pone.0019725.s003.tif]

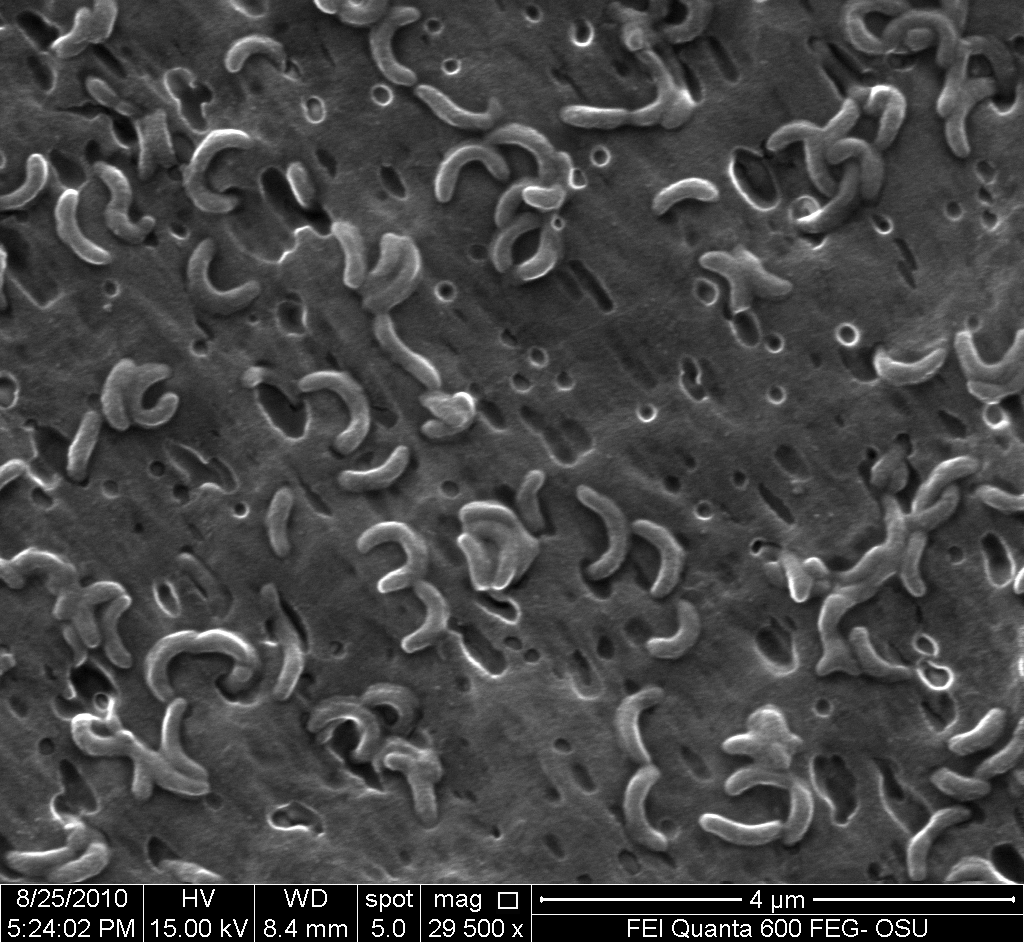

Supplement: Figure S4 — Original picture taken with the Scanning Electron Microscope utilized to create Figure 2 . 2A. Cells after 2 days in stationary phase in the light (70 µmol photons m−2 s−1). 29500× magnification. (TIF) [file pone.0019725.s004.tif]

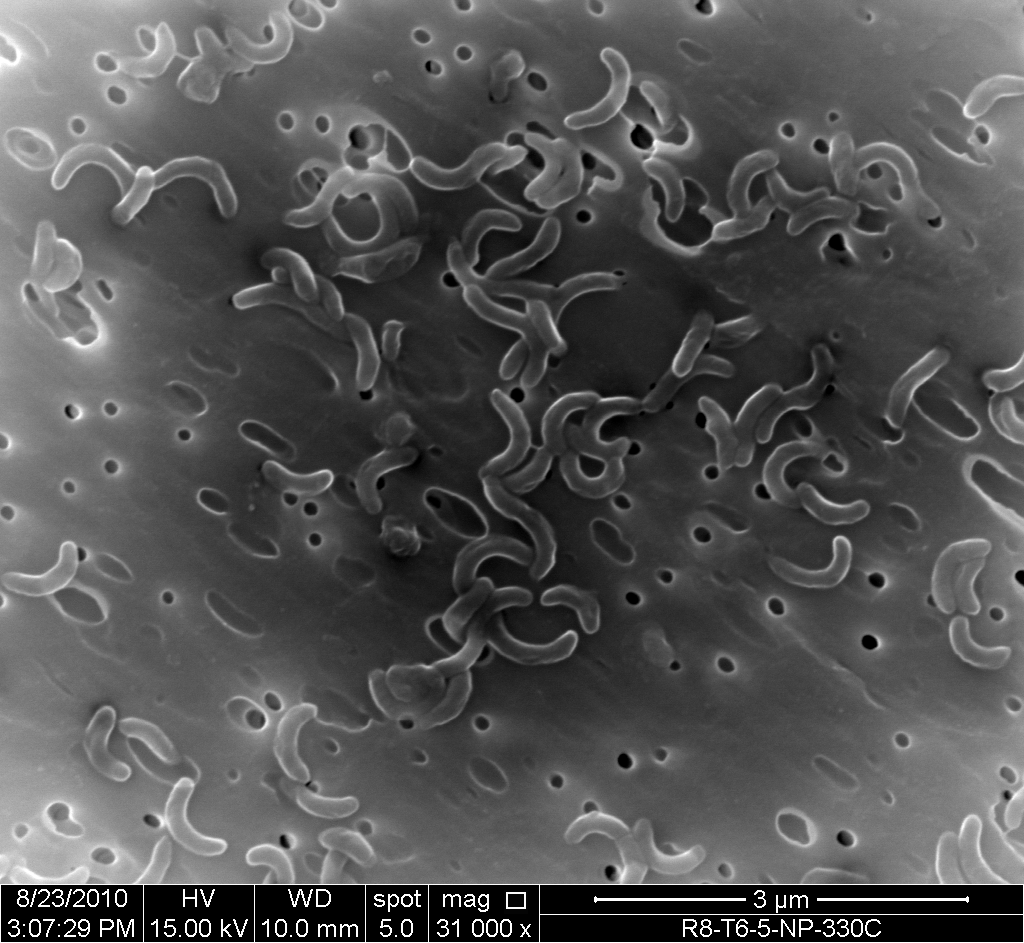

Supplement: Figure S5 — Original picture taken with the Scanning Electron Microscope utilized to create Figure 2 . 2B. Cells after 5 days in stationary phase in the light (70 µmol photons m−2 s−1). 31000× magnification. (TIF) [file pone.0019725.s005.tif]

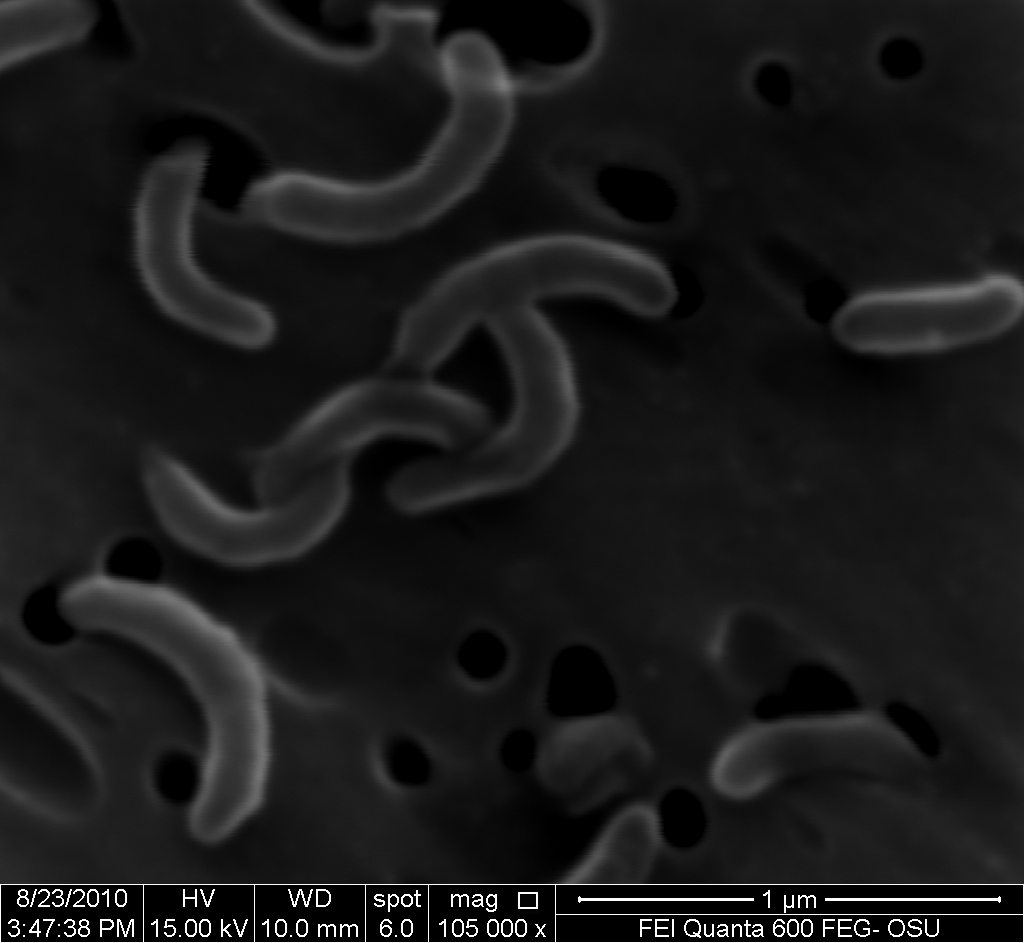

Supplement: Figure S6 — Original picture taken with the Scanning Electron Microscope utilized to create Figure 2 . 2C. Cells after 5 days in stationary phase in the light (70 µmol photons m−2 s−1). 105000× magnification. (TIF) [file pone.0019725.s006.tif]

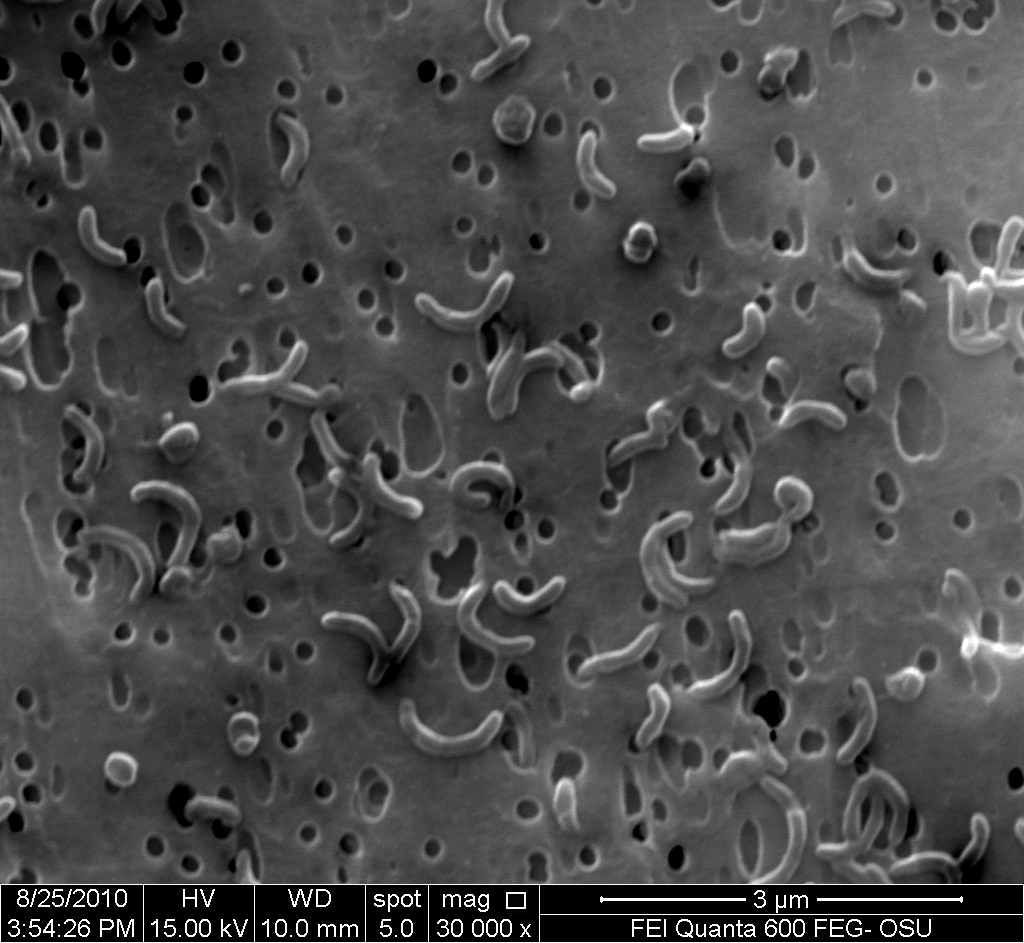

Supplement: Figure S7 — Original picture taken with the Scanning Electron Microscope utilized to create Figure 2 . 2D. Cells after 8 days in stationary phase in the light (70 µmol photons m−2 s−1). 30000× magnification. (TIF) [file pone.0019725.s007.tif]

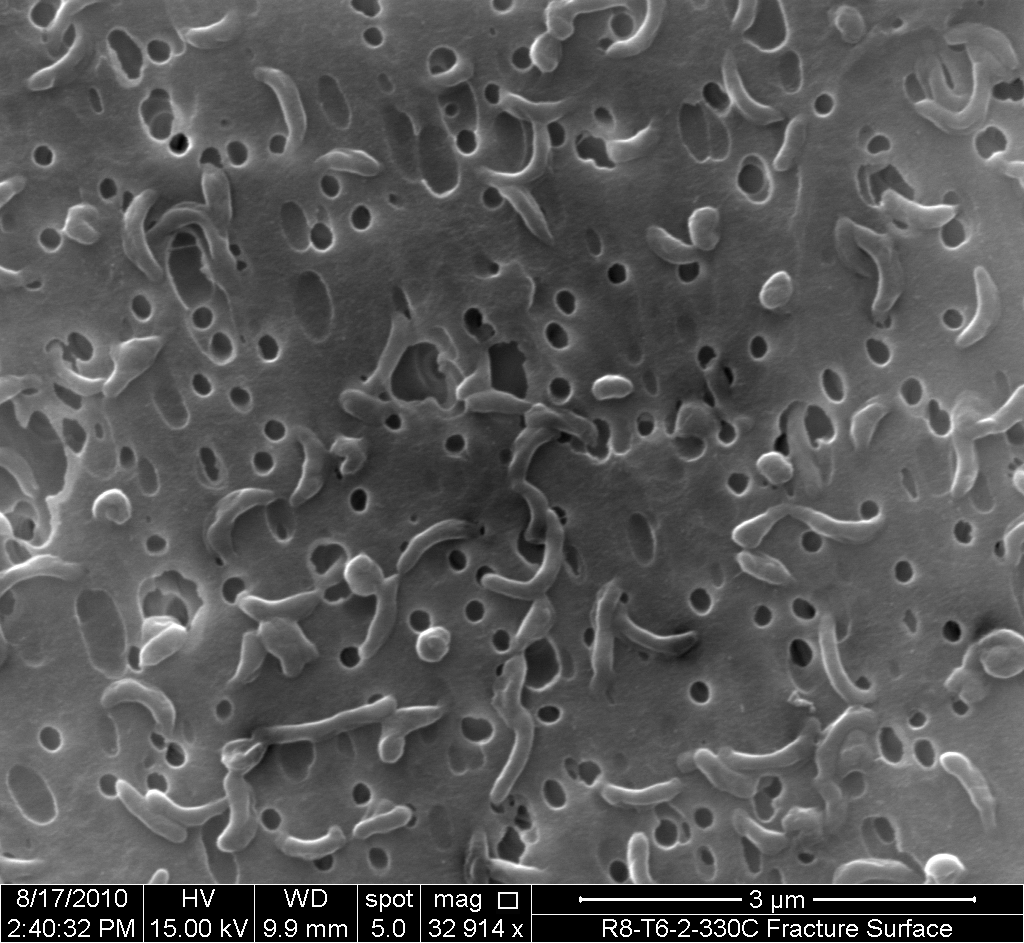

Supplement: Figure S8 — Original picture taken with the Scanning Electron Microscope utilized to create Figure 2 . 2E. Cells after 2 days in stationary phase in the dark. 33000× magnification. (TIF) [file pone.0019725.s008.tif]

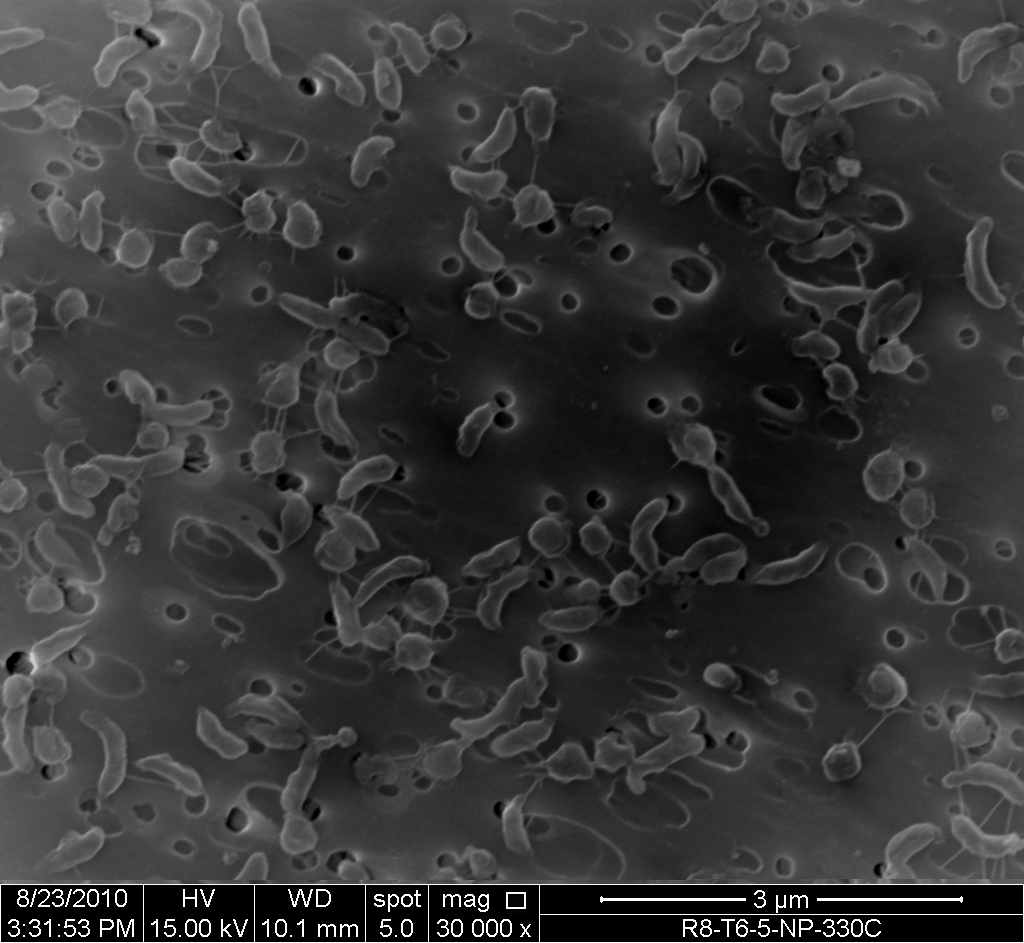

Supplement: Figure S9 — Original picture taken with the Scanning Electron Microscope utilized to create Figure 2 . 2F. Cells after 5 days in stationary phase in the dark. 30000× magnification. (TIF) [file pone.0019725.s009.tif]

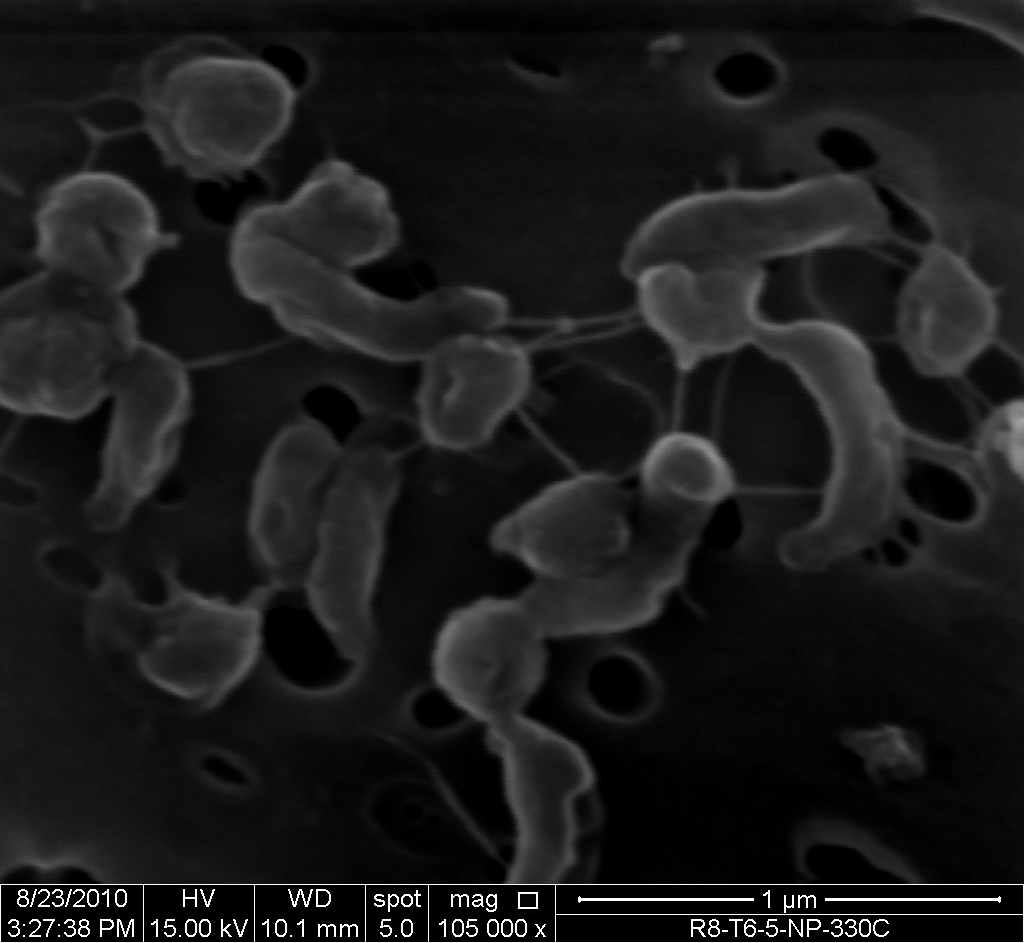

Supplement: Figure S10 — Original picture taken with the Scanning Electron Microscope utilized to create Figure 2 . 2G. Cells after 5 days in stationary phase in the dark. 105000× magnification. Pili connecting cells can be observed. (TIF) [file pone.0019725.s010.tif]

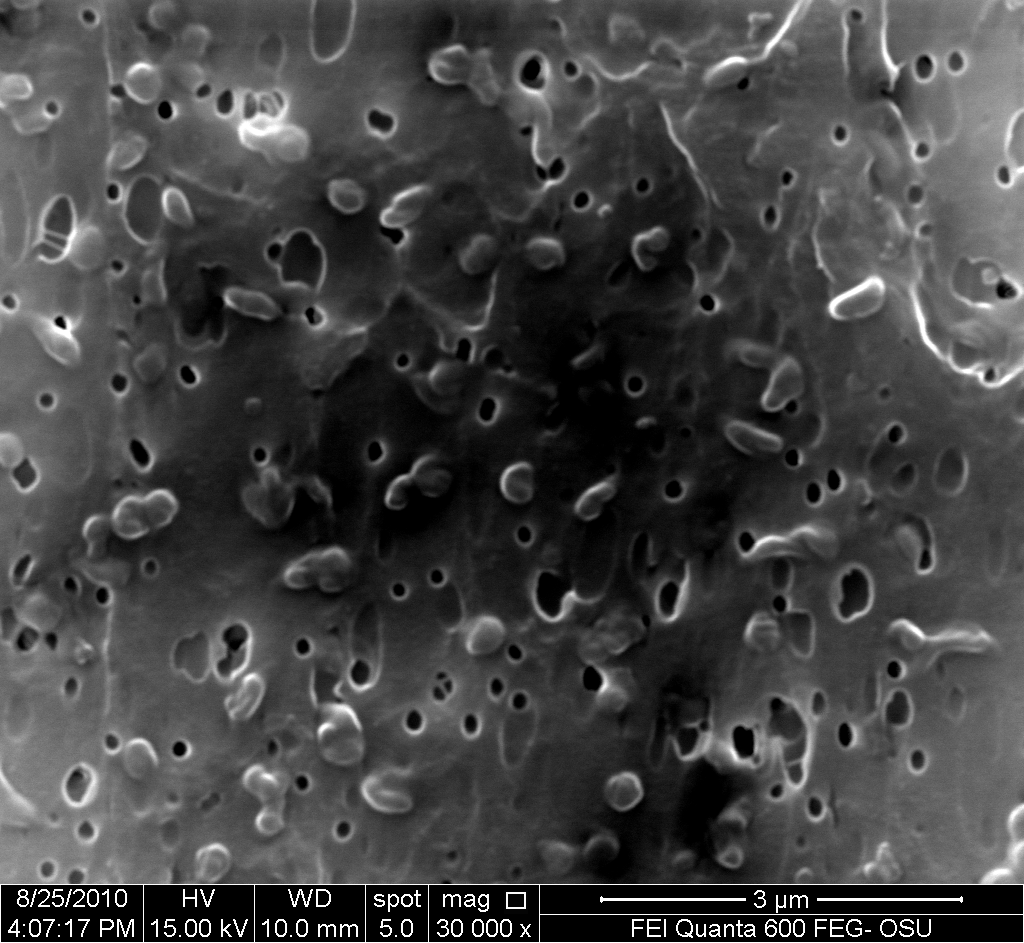

Supplement: Figure S11 — Original picture taken with the Scanning Electron Microscope utilized to create Figure 2 . 2H. Cells after 8 days in stationary phase in the dark. 30000× magnification. (TIF) [file pone.0019725.s011.tif]
