# Supplementary material for: Energy Starved Candidatus Pelagibacter Ubique Substitutes Light-Mediated ATP Production for Endogenous Carbon Respiration
Source: PLoS One. 2011 May 9;6(5):e19725. doi: 10.1371/journal.pone.0019725 (PMC3090418; doi:10.1371/journal.pone.0019725)
Supplement: Table S1 — A) Genes upregulated in cells grown in light∶dark 12∶12 hrs cycles (light treatment). B) Genes upregulated in cells grown in constant darkness (dark treatment). Differential expression between treatments was considered biologically relevant for ≥2 fold change for single genes and ≥1.7 fold change for potential operons (t-test, p≤0.05). Genes were considered potential operons if they were consecutive with no (or very small) intergenic space and were either all up or down regulated under light or dark treatment. Question marks (?) represent unknown gene functions. Genes IDs refer to GenBank, SAR11_ #### locus tag is given for unknown genes. (DOC) [file pone.0019725.s012.doc]

A) Genes up-regulated in the light treatment

| ***Gene ID*** | ***Gene(s)*** | ***Predicted gene product(s)/function*** | ***Fold change***  ***(mean+s.d.)*** |
| --- | --- | --- | --- |
| ***Potential operons:*** |  |  |  |
| 3516571-3 | SAR11_0964-5*, yiaK* | transcription regulator (Fur), ?, YiaK | 7.4+0.5 |
| 3517728-9 | *rbr, glpG* | rubrerythrin, glycerol-3-phosphate dehydrogenase subunit C | 6.0+1.3 |
| 3517610-16 | *ccmB, ccmC, ccmD,ccmE,*  *ccmF, ccmG,*  *ccmH* | cytochrome *c* biogenesis proteins | 4.4+1.6 |
| 3517607-8 | *aspC, ildD* | transaminase, lactate-dehydrogenase | 3.7+1.2 |
| 3517096-8 | *fbaB, pgk, gap* | glycolosis | 3.5+0.8 |
| 3517602- 6 | SAR11_0274*, gyaR, glcF, glcE, glcD* | genes related to the glyoxylate cycle | 3.2+0.3 |
| 3517869-70 | SAR11_1163-4 | ? | 3.1+0.3 |
| 3516665-6 | *vacJ, yrbC* | ?, transporter | 2.8+0.6 |
| 3517422-3 | SAR11_1022-3 | permease, ? | 2.6+0.4 |
| 3517234-6 | SAR11_0471*, dcd, murA* | ?, deoxycytidine triphosphate deaminase, UDP-N-acetylglucosamine 1-carboxyvinyltransferase | 2.6+0.4 |
| 3517867-8 | SAR11_1161-2 | ? | 2.5*+*0.3 |
| 3517595-9 | *msmX, msmX2, ycjP,* SAR11_0270-71 | ABC transporters | 2.4+0.6 |
| 3517863-4 | *nrdB, nrdA* | ribonucleoside-diphosphate reductase | 2.2+0.9 |
| 3517342-4 | *tatA, tatB, tatC* | transport of proteins across cytoplasmic membrane | 2.1+0.5 |
| 3517114-5 | *folP, mrsA* | vitamin metabolism, glycolisis/gluconeogenesis | 2.1+0.3 |
| ***Single genes:*** |  |  |  |
| 3517456 | *xerD* | recombination and DNA repair | 6.6 |
| 3517798 | SAR11_1242 | putative transcription regulator | 5.7 |
| 3517862 | SAR11_1157 | putative methyltransferase | 5 |
| 3517429 | *metY* | O-acetylhomoserine (thiol)-lyase | 3.8 |
| 3517588 | SAR11_0259 | ? | 3.7 |
| 3516867 | *recA* | recombination and DNA repair | 3.4 |
| 3517087 | *aroC* | chorismate synthase | 3.4 |
| 3516926 | *ibpA* | heat shock protein | 2.9 |
| 3517600 | *adhP* | alcohol dehydrogenase | 2.7 |
| 3517455 | SAR11_1018 | putative methyltransferase | 2.6 |
| 3516869 | *alaS* | alanine-tRNA ligase | 2.6 |
| 3516576 | *dgtP* | dGTPase | 2.6 |
| 3516875 | *guaB* | IMP dehydrogenase | 2.4 |
| 3517130 | *suhB* | extragenic suppressor protein suhB | 2.3 |
| 3516818 | SAR11_0748 | tRNA synthesis | 2.1 |
| 3516752 | SAR11_0138 | transcriptional regulator, MarR family | 2.1 |
| 3516577 | *argS* | arginine-tRNA ligase | 2 |
| 3517361 | *miaA* | tRNA isopentenyltransferase | 2 |
| 3517457 | SAR11_1020 | Putative acetyltransferase | 2 |

**B) Genes up-regulated in the dark treatment**

| ***Gene ID*** | ***Gene(s)*** | ***Predicted gene product(s)/function*** | ***Fold change***  ***(mean+s.d.)*** |
| --- | --- | --- | --- |
| ***Potential operons:*** |  |  |  |
| 3516594-5 | *fbcH, petB* | ubiquinol-cytochrome-*c* reductase: cytochrome *c1*, cytochrome *b*. | 2.6+0.8 |
| 3516722-3 | *tpa, ald* | taurine-pyruvate aminotransferase, alanine dehydrogenase | 2.6+0.4 |
| 3516888-9 | *groES, groEL* | protein folding and stabilization under stress | 2.3+0.3 |
| 3517493-8 | SAR11_1305*, asnB,* SAR11_1307*, gltB2,* SAR11_1309*, amt* | glutamine synthetase III – like, glutamine amidotransferase subunit, GXGXG motif, glutamate synthase large subunit, ?, ammonium transporter | 2.2+0.2 |
| 3516768-70 | *atpB, atpE, atpB* | H+-transporting two-sector ATPase subunits: A, C, b1 | 2+0.5 |
| 3517474-5 | *rpoC, rpoB* | RpoC and RpoB, maintenance of the transcription bubble, and positioning of the nascent RNA strand | 2+0.3 |
| 3516710-1 | *aprB, aprA* | adenylyl-sulfate reductase chains B and A | 1.9­+0.3 |
| 3516962-3  3516561-2 | *yhdW, yhdX, yhdY, yhdZ* | amino acid ABC transporter | 1.9+0.4 |
| 3517558-61 | *atpD, atpG, atpA, atpH* | F1-ATP Synthase Beta Chain, H+-transporting two-sector ATPase: gamma, alpha and delta chains | 1.9+0.3 |
| 3517023-4 | *smoM,* SAR11_0865 | periplasmic mannitol-binding protein, TRAP-type mannitol/chloroaromatic compound transport system | 1.8+0.3 |
| 3517259-60, 3517299-300, 3517302, 3517304, 3517307, 3517310-1, 3517314, 3517316-8, 3517476-7, 3517479 | *rplO, rpsE, rplF, rpsH, rplE, rplN, rplP, rpsS, rplB, rplC, fusA, rpsG, rpsL, rplL, rplJ, rplK* | ribosomal proteins and elongation factors | 1.9+0.2 |
| 3516747-9 | *ctaB, cox1* | cytochrome *c* oxidase assembly factor, cytochrome-*c* oxidase, chain I | 1.7+0.2 |
| ***Single genes:*** |  |  |  |
| 3516825 | *aldB* | acetaldehyde dehydrogenase II | 3.5 |
| 3517483 | *tufB* | translation elongation factor EF-Tu | 3.3 |
| 3517407 | SAR11_0765 | ? | 3 |
| 3516721 | *tauA* | taurine transport system periplasmic protein | 2.9 |
| 3516785 | SAR11_0794 | ? | 2.8 |
| 3517459 | *livJ2* | Leu/Ile/Val-binding protein precursor | 2.8 |
| 3517439 | SAR11_1040 | H+ translocating pyrophosphate synthase | 2.5 |
| 3517043 | *livJ* | leucine-, isoleucine-, valine-, threonine-, and alanine-binding protein precursor | 2.3 |
| 3517646 | *yjcG* | Na+/solute symporter | 2.3 |
| 3517481 | *secE* | protein secE/sec61-gamma protein | 2.3 |
| 3516629 | *pilA* | Pilin | 2.2 |
| 3516881 | *braC* | putative Leu/Ile/Val-binding protein | 2.2 |
| 3517411 | SAR11_0769 | putative binding protein component of ABC sugar transporter | 2.2 |
| 3517062 | *pr* | proteorhodopsin | 2.1 |
| 3517489 | *opuAC* | Substrate-binding region of ABC-type glycine betaine transport system | 2.1 |
| 3517594 | SAR11_0266 | TRAP dicarboxylate transporter - DctP subunit | 2.1 |
| 3517089 | *ispB* | octaprenyl-diphosphate synthase | 2.1 |
| 3517388 | *glyA* | glycine hydroxymethyltransferase | 2.1 |
| 3517354 | *ppiB* | peptidylprolyl isomerase | 2.1 |
| 3517254 | *rpoA* | DNA-directed RNA polymerase | 2 |
| 3516637 | *hflC* | proteolytic degradation | 2 |
