# Supplementary material for: Energy Starved Candidatus Pelagibacter Ubique Substitutes Light-Mediated ATP Production for Endogenous Carbon Respiration
Source: PLoS One. 2011 May 9;6(5):e19725. doi: 10.1371/journal.pone.0019725 (PMC3090418; doi:10.1371/journal.pone.0019725)
Supplement: Table S2 — A) Genes upregulated in cells grown in light∶dark cycles (light treatment). B) Genes upregulated in cells from the light treatment transferred to darkness for 6 hours (light-to-dark treatment). Differential expression between treatments was considered biologically relevant for ≥2 fold change for single genes and ≥1.7 fold change for potential operons (t-test, p≤0.05). Genes were considered potential operons if they were consecutive with no (or very small) intergenic space and were either all up or down regulated under light or dark treatment. Question marks (?) represent unknown gene functions. Genes IDs refer to GenBank, SAR11_ #### locus tag is given for unknown genes. (DOC) [file pone.0019725.s013.doc]

A) Genes up-regulated in the light treatment

| ***Gene ID*** | ***Gene(s)*** | ***Predicted gene product(s)/function*** | ***Fold change***  ***(mean+s.d.)*** |
| --- | --- | --- | --- |
| ***Potential operons:*** |  |  |  |
| 3516571-2 | SAR11_0964-5 | transcription regulator (Fur), ? | 2.7+0.0 |
| 3517728-9 | *rbr, glpG* | rubrerythrin, glycerol-3-phosphate dehydrogenase subunit C | 1.7+0.0 |

B) Genes up-regulated in the light-to-dark treatment

| ***Gene ID*** | ***Gene(s)*** | ***Predicted gene product(s)/function*** | ***Fold change***  ***(mean+s.d.)*** |
| --- | --- | --- | --- |
| ***Single genes:*** |  |  |  |
| 3516731 | *amtB* | ammonium transporter | 2 |
